# Severe pneumonia with formation of a pulmonary cavity associated with long-term rituximab therapy in multiple sclerosis

**DOI:** 10.1186/s42466-020-00074-0

**Published:** 2020-10-12

**Authors:** Carolin Schwake, Ralf Gold

**Affiliations:** Department of Neurology, St. Josef-Hospital, Ruhr-University Bochum, Gudrunstr. 56, 44791 Bochum, Germany

**Keywords:** Multiple sclerosis, Rituximab, Pneumonia, Pulmonary cavity

## Abstract

Nowadays B-cell depletion via anti-CD20 antibodies is commonly applied in the treatment of multiple sclerosis (MS). Yet, not much is known about infection risks associated with long-term B-cell depletion in the specific context of MS.

We present the case of a 45-year-old male patient who developed severe pneumonia following 6 years of rituximab treatment for highly active relapsing-remitting MS. The patient had no additional chronic disease as well as no history of foreign travel. Although the unusual formation of a pulmonary cavity raised suspicion for tuberculosis, repeated testing via bronchoscopy and sputum remained negative. Prolonged antibiotic therapy with piperacillin/tazobactam and amoxicillin/ clavulanate led to complete recovery from symptoms.

This case shows the potential risk of serious infections following continuous B-cell depletion in MS and illustrates the importance of future vigilance.

## Introduction

Initially emerging from the therapy of B-cell neoplasms, the mouse/human chimeric anti-CD20 antibody rituximab opened new pathways in treatment of autoimmune disease. Off-label use of rituximab was shown to be effective and well-tolerated in relapsing-remitting multiple sclerosis (RRMS) [1]. Nevertheless, data on the safety of long-term rituximab administration in MS are scarce.

We herein report the case of a 45-year-old male patient who developed severe pneumonia with formation of a pulmonary cavity following 6 years of rituximab treatment for highly active RRMS.

## Case report

The patient presented to our outpatient clinic for the first time in 2014. He had a history of previously diagnosed, untreated RRMS and suffered from brain stem symptoms with vertical nystagmus and loss of visual acuity. Because of high clinical and gadolinium-enhancing activity B-cell depleting therapy with rituximab was implemented. The patient received regular infusions of rituximab (500 mg) every six to eight months. Treatment intervals were individualized based on CD19+ B-cell monitoring. Over the time span of 6 years the patient suffered from trigeminal neuralgia and an increasing gait ataxia, but overall his clinical condition remained relatively stable (EDSS 3,5). He had no other chronic disease and no history of foreign travel.

**Fig. 1:**
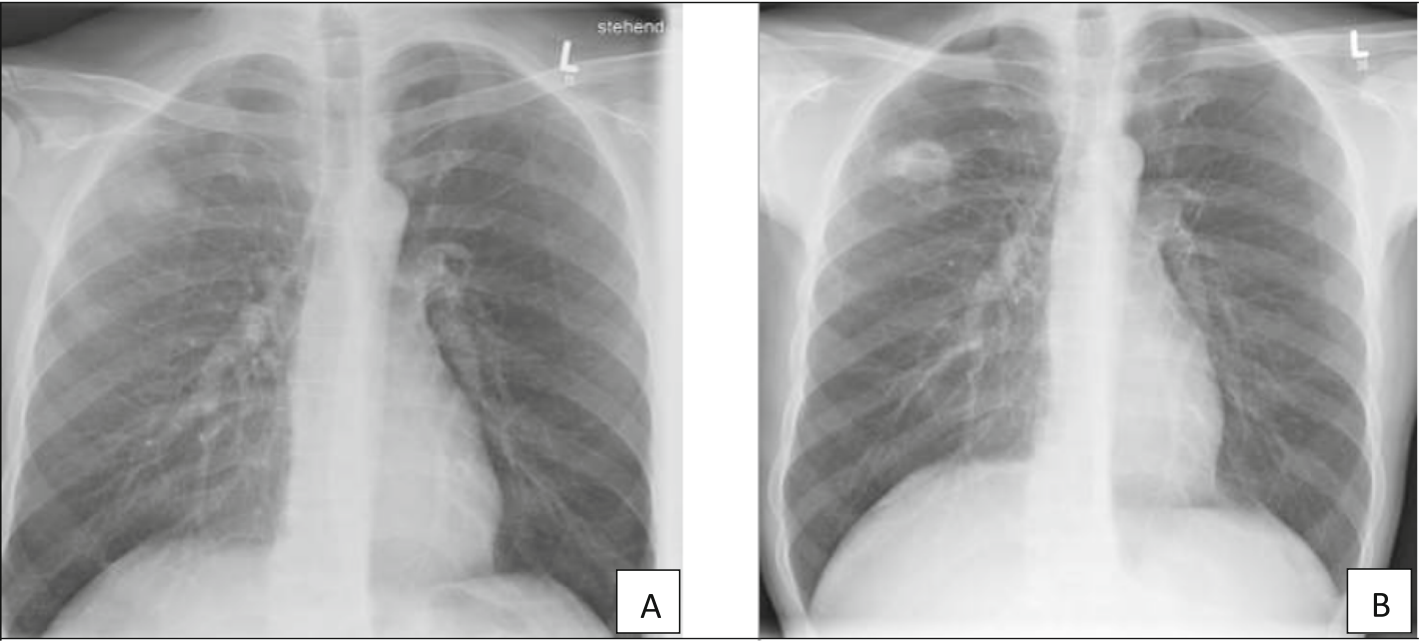
chest X-rays performed in January (**a**) and February (**b**) documenting the formation of a cavitary lung lesion over the time span of 6 weeks

**Fig. 2:**
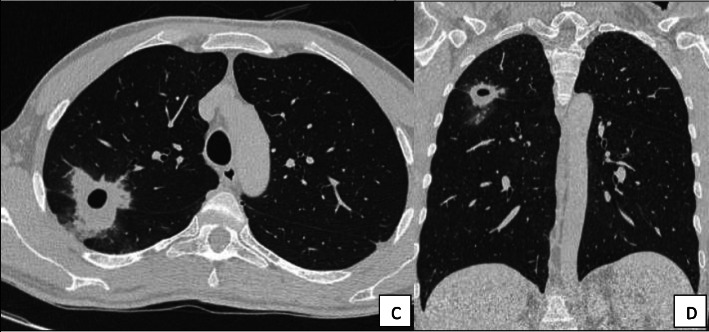
Involvement of the right upper and inferior lung lobe shown in chest CT in transverse (**c**) and coronal view (**d**)

In January 2020 the patient developed progressive weakness and night sweats. Because of a productive cough, chest x-ray was performed and revealed an infiltration of the right upper lung. Empiric antibiotic treatment with ampicillin/sulbactam was prescribed for 7 days for community acquired pneumonia. However, 6 weeks later, another x-ray documented the formation of a cavitary lesion **(**Fig. 1**)**. A computed tomography of the chest revealed engagement of the upper and inferior lobe of the right lung **(**Fig. 2**)**. Blood testing showed an elevation of leukocytes (12,200 leukocytes/μL, 83% neutrophils) and CRP (68 mg/L). Consequently, the patient was admitted to the hospital for extended diagnostic work-up. A transbronchial biopsy verified granulocytic inflammation. No evidence of malignancy was found. Repeated testing for tuberculosis, fungi and bacteria remained all negative. Intravenous antibiotic therapy with piperacillin/tazobactam was given for 12 days followed by oral treatment with amoxicillin/ clavulanate for another 7 days. Therapy resulted in gradual clinical improvement and regression of CRP values.

Further immunological evaluation demonstrated complete depletion of CD19+ B-cells. For the first time, the serum IgG level was analyzed and shown to be slightly decreased (IgG 6.30 g/L, normal range 7.0–16-0 g/L).

## Discussion

Over a decade ago, the 48-week phase II HERMES trial for RRMS showed a similar incidence of infections in patients treated with rituximab (69,6%) and placebo (71,4%). Since then, the safety profile of B-cell depleting therapy with rituximab in MS is still of great interest.

Two large Swedish studies provide more recent data and estimate an overall higher infectious risk in this context. A retrospective observational multicenter study by Salzer et al. (2016) identified infections as most common non infusion related adverse events [2]. Moreover, Luna et al. (2019) came to the conclusion that rituximab led to the highest rate of serious infections in MS therapy (incidence rate 19,7 per 1000 person-years) as compared to fingolimod (14,3), natalizumab (11,4) and injectables (8,9) [3]. Mean duration of rituximab treatment in these studies was 21,6 months [2], respectively 2.0 patient-years [3].

It remains unclear if B-cell depleting therapy for MS increases infectious complications over time. We here report an interesting case of an otherwise healthy individual who developed severe pneumonia after 6 years of long-term rituximab therapy.

Analysis of long-term rituximab treatment (36 months to 7 years) in different auto-immune neurological disorders revealed no particular serious adverse events. Yet, only 29 patients were included in this study [4]. On the other hand, rituximab maintenance therapy was shown to increase infection risks as a result of neutropenia and hypogammaglobulinemia in hematological [5], rheumatological or auto-immune disorders [6].

In a cohort of 50 NMOSD patients hypogammaglobulinemia occurred in 64% after long-term rituximab treatment and led to severe infections in 5 cases. In contrast to our patient, IgG levels were found to be much lower (3.1 to 5.1 g/L) [7].

Another noteworthy aspect is the formation of a complicating pulmonary cavitary lesion. Suspicion of an underlying opportunistic agent was high. According to the literature, infections with *Pneumocystis jirovecii* associated to rituximab are rare if CD20 B-cell depletion is the only cause for immunosuppression [8]. Tbc re-activation under rituximab may occur [9], but it is still under discussion to what extent. Pulmonary nocardiosis sometimes can mimic tuberculosis in immunosuppressed patients [10]. Yet, diagnostic work-up (including invasive sampling) did not provide any pathogen identification in our patient and only prolonged antibiotic treatment eventually led to gradual improvement.

For conclusion, further attention to evaluate long-term safety of B-cell depletion in MS treatment is needed.

## Data Availability

Not applicable.

## Abbreviations

CRP: C-reactive protein
EDSS: Expanded disability Status Scale
HERMES: Helping to Evaluate Rituxan in Relapsing–Remitting Multiple Sclerosis
HIV: Human acquired immunodeficiency virus
NMOSD: Neuromyelitis optica spectrum disorders
RRMS: Relapsing-remitting Multiple Sclerosis
